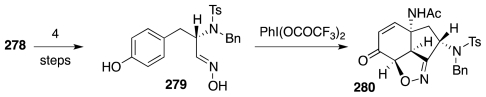# The Chemical Synthesis of Tetrodoxin: An Ongoing Quest

**DOI:** 10.3390/md9102046

**Published:** 2011-10-20

**Authors:** Jaclyn Chau, Marco A. Ciufolini

**Affiliations:** Department of Chemistry, University of British Columbia, 2036 Main Mall, Vancouver, BC V6T 1Z1, Canada; E-Mail: jchau@chem.ubc.ca

**Keywords:** tetrodotoxin, synthesis, nitrenes, hypervalent iodine compounds

## Abstract

This contribution reviews all the synthetic work on tetrodotoxin that has appeared in the literature through June 2011.

## 1. Introduction

**Figure 1:**
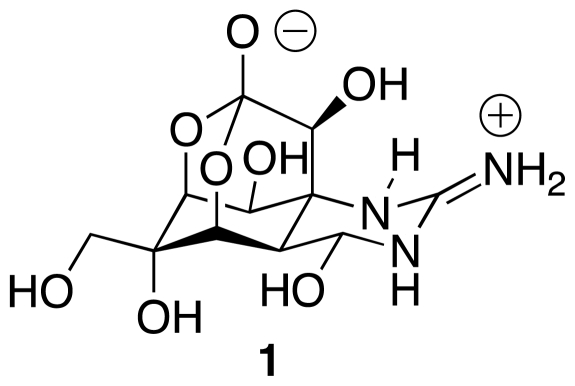
Chemical structure of tetrodotoxin (TTX).

This contribution reviews the literature on the synthesis of tetrodotoxin (TTX, **1**, Figure 1), through the first half of 2011 [1]. This substance, the neurotoxic principle of the formidable *Tetraodon* fish (puffer fish, fugu), is one of the classical targets in synthetic organic chemistry. Its delightfully intricate architecture captured the imagination of the synthetic community as soon after its structure was determined independently, but nearly simultaneously, by Woodward [2], Goto [3], Tsuda [4], and respective collaborators, in the mid-1960s. Efforts to conquer TTX reached a climax in 1972, when Kishi and coworkers announced their history-making first synthesis of **1**.

Over the following quarter-century, the synthetic community showed little interest in TTX, but the past decade has witnessed a resurgence of activity in this area, fueled by in part by landmark syntheses by Isobe and DuBois in the early 2000s. Synthetic research toward **1** continues unabated to this day.

But why pursue yet another synthesis of tetrodotoxin, despite all the brilliant work that has already been reported?

The reasons are manifold. At an academic level, the structure of **1** lends itself beautifully to the exploration of uncharted approaches and it provides an unforgiving platform to evaluate new chemical transformations. Thus, any new methodology or strategy that translates into a victorious assault on **1** must necessarily be significant. For instance, the formation of C–N bonds via nitrene insertion into C–H linkages [5] was a concept largely confined to physical organic research [6] prior to 2003, despite our early studies in this area [7–11]. One could argue that the credibility of such a transformation as a synthetically useful resource increased dramatically after DuBois employed a technologically advanced form thereof in his synthesis of TTX (*vide infra*). In a like manner, a strategic principle that facilitates or ameliorates the synthesis of **1** necessarily advances the state of the art, and in all likelihood it will be incorporated rapidly into the canon of contemporary synthetic methodology.

There are also significant practical reasons for engaging in the above pursuit. A sound, concise avenue to TTX is key to medicinal chemistry research that could identify a congener of the natural product that might be useful in human medicine. Briefly, the bioactivity of TTX is ascribed to potent inhibition of voltage-gated Na^+^ channels. Blocking such channels in a controlled fashion appears to be desirable in the treatment of conditions such as Parkinson’s disease [12] and chronic pain in terminally ill cancer patients [13]. Tetrodotoxin is too hazardous to be used as a drug, but analogs with reduced toxicity and better pharmacological profile may well become viable medicaments.

## 2. Strategies Explored in Connection with Synthetic Work on TTX

**Scheme 1:**
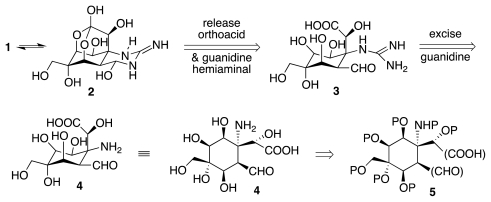


Central to all published work in the tetrodotoxin area is the retrosynthetic logic outlined in Scheme 1. Consider the electrostatically neutral form of the natural product, **2**. Release of orthoacid and guanidine hemiaminal units reveals a highly substituted cyclohexane **3**, which may be further simplified to **4** by excision of the guanidine segment. Thus, any synthesis of **1** must produce structure **4** or an equivalent thereof, such as **5**, wherein the substituents in parentheses represent latent forms of the requisite functionalities, and units P stand for appropriate protecting groups.

**Scheme 2:**
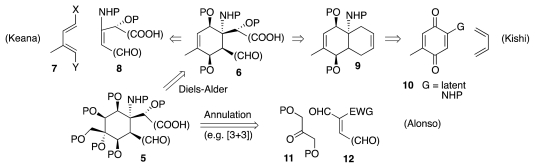


Recorded efforts aiming to reach **5** may be grouped into three categories, depending on the strategy that was employed to control the relative configuration of the substituents that adorn the cyclohexane core. Simplification of **5** by removal of some of the oxygen functionalities produces cyclohexene **6** (Scheme 2), the double bond of which enables oxygenation of the exocyclic methyl group by allylic oxidation and of the olefinic carbons by epoxidation or osmylation. A plausible route to **6** envisions a Diels-Alder reaction of diene **7** with a dienophile of the type **8**. Such is in fact the approach explored by Keana. A subtler analysis leads to bicyclic retron **9**, which emerges upon reconnecting the carboxy and formyl groups in **6**. Compound **9** is the Diels-Alder adduct of quinone **10** with butadiene. The landmark Kishi synthesis of **1** rested indeed upon this logic. Compound **5** is also available by a variety of annulation reactions. Indeed, a [3 + 3] annulation that entails the union of fragments **11** and **12** was researched by Alonso.

**Scheme 3:**
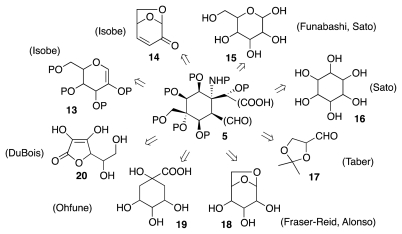


Alternative routes to **5** may involve the elaboration of a highly oxygenated, naturally occurring material such as a sugar, a cyclitol, or a related substance (Scheme 3). Strategies relying upon this principle are apparent in the work of Isobe (use of **13** and **14**), Funabashi (**15**), Sato (**15**, **16**), Taber (**17**), Fraser-Reid (**18**), Alonso (**18**), Ohfune (**19**), and DuBois (**20**).

**Scheme 4:**
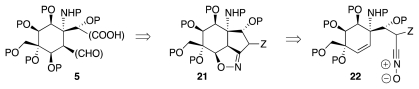


A third possibility emerges upon recognition that the *syn* relative configuration of the acetic acid branch, the formyl group and its vicinal oxygen functionality in **5** enable the control of relative configuration through the fragmentation of an isooxazoline such as **21** (Z = functional group that facilitates fragmentation), which in turn could result of via an intramolecular nitrile oxide cycloaddition (INOC) reaction (Scheme 4). Approaches to **1** described by Fukuyama and by us rely indeed on this logic.

This review illustrates salient aspects of the foregoing efforts, starting with a summary of the reported total syntheses of **1**. This shall be followed by a discussion of the various synthetic studies disclosed in the primary literature [14] as of this writing.

## 3. The Diels-Alder Route to Tetrodotoxin

**Scheme 5:**
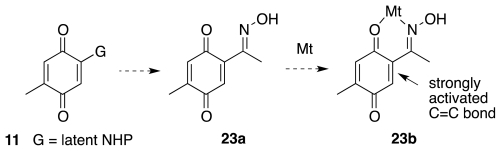


As indicated earlier, the Kishi synthesis of TTX [15–18] evolved from a Diels-Alder reaction of quinone **11**. Group G in **11** may not be an actual or protected amino residue, because the electron-donating nature of either would hamper the cycloaddition step. Instead, G must be an electron-withdrawing group that could later be advanced to a protected amine. The choice fell on oximino quinone **23a**. The oxime segment would direct the Diels-Alder reaction to the correct double bond, especially upon coordination with a chelating Lewis acid (cf. Mt in **23b**, Scheme 5), and ultimately undergo Beckmann rearrangement to install the requisite nitrogen functionality. An enantioselective Diels-Alder reaction was well beyond the scope of early 1970s technology. Consequently, the initial cycloadduct was obtained as the racemate, and the overall synthesis produced (±)-**1**.

**Scheme 6:**
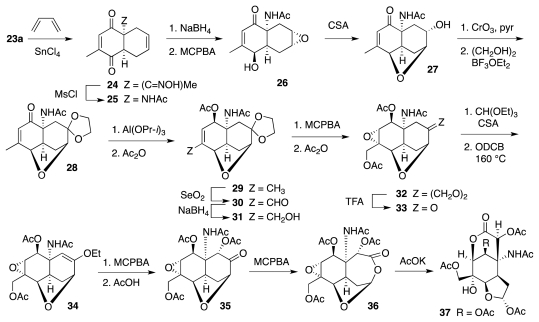


The opening moves of the synthesis appear in Scheme 6. Regioselective Diels-Alder reaction of **23a** with butadiene promoted by SnCl_4_ afforded **24**, which upon *O*-mesylation of the oxime underwent transposition to **25**. Substrate-controlled regio- and stereoselective carbonyl reduction and olefin epoxidation produced **26**, which was advanced to **27** as a prelude to oxidative upgrading of the erstwhile quinoid nucleus and oxidative cleavage of the right-hand side ring. Key steps in this phase of the synthesis were the stereoselective acetoxylation of **34** initiated by epoxidation of the vinyl ether, the regioselective Bayer-Villiger oxidation of ketone **35** directed by the ether oxygen, and the tandem lactone opening/intramolecular epoxide cleavage leading to **37**.

**Scheme 7:**
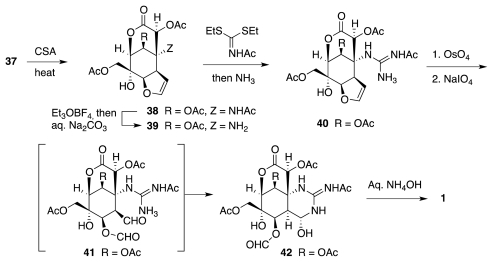


The assembly of racemic tetrodotoxin was completed from **37** as shown in Scheme 7. Elimination of acetic acid from the tetrahydrofuran moiety of **37** provided dihydrofuran **38**, which upon *N*-guanidylation yielded **40**. Lemieux-Johnson oxidative cleavage of the double bond unveiled aldehyde **41**, which instantly cyclized to form **42**. Finally, release of formyl and acetyl groups occurred with concomitant (and anticipated [2]) orthoacid formation to deliver fully synthetic **1**.

Overall, the synthesis was accomplished in a noteworthy 28 linear steps from **11**. Even by taking into account the 3 steps required to prepare **11**, this route to TTX is remarkably concise, especially when one considers that the status of the chemical technology of the early 1970s. The fact that only 5 steps involved *bona fide* protecting group manipulations is an eloquent testimony to Kishi’s masterful planning. Indeed, forty years after its publication, this masterpiece of synthetic chemistry remains the yardstick against which all other efforts are evaluated.

## 4. Syntheses of TTX from Carbohydrates and Congeners

**Scheme 8:**
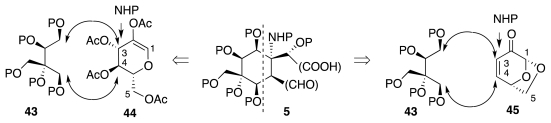


The total synthesis of natural products from carbohydrate building blocks [19–35] became an exceedingly active area of research in the late 1970s. Since then, countless targets have fallen to carbohydrate-based efforts, proving beyond doubt the validity of such an approach to chemical synthesis. An especially significant achievement in this area is the first ever enantioselective synthesis of tetrodotoxin by Isobe and collaborators. In fact, these workers described two total syntheses of (−)-**1**: the first one (2003) from glucal **44** [36] and the second one (2004) from levoglucosenone, **45** [37,38] (Scheme 8). In either case, the key objectives were the annulation of the cyclohexane ring of **5** onto C-3 and C-4 of the glucose derivative, and the installation of the nitrogenous functionality at C-3.

**Scheme 9:**
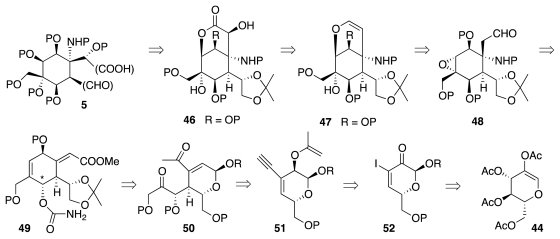


Scheme 9 delineates key strategic aspects of the 2003 synthesis. A great deal of background work [39–45] indicated that α-hydroxylactone **46** could be made from vinyl ether **47**, which in turn would result from base-promoted cyclization of epoxyaldehyde **48**. Among the numerous methods explored for the introduction of the nitrogen functionality, only the Michael-type cyclization of carbamate **49** performed adequately. A drawback of this approach is that the configuration of the starred carbon in **49** must be opposite that required for the natural product, necessitating an inversion of configuration at a later juncture. It was further envisioned that **49** would derive from **50** by aldol-dehydration and reduction. The final correlation with **44** envisaged introduction of the acetonyl segment through Claisen rearrangement, and of the conjugated methyl ketone by hydration of an alkyne. This led to retrosynthetic intermediate **51**, which can be readily made from **44** via **52**.

**Scheme 10:**
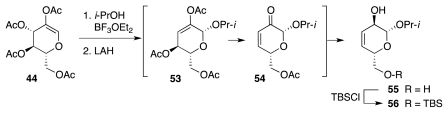


**Scheme 11:**
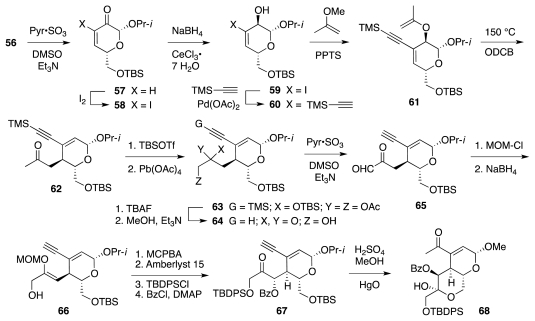


**Scheme 12:**
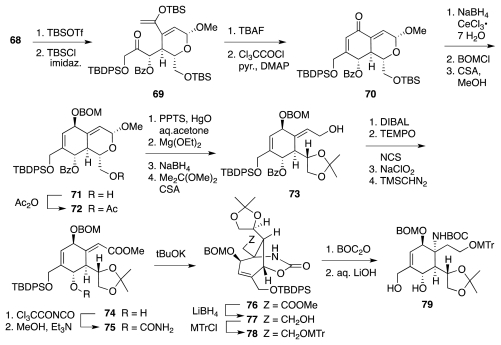


The synthesis began with a straightforward sequence that produced **56** from **44** (Scheme 10), in turn available from glucose in 3 steps. Material **56** was then elaborated to vinyl ether **61** (Scheme 11), which underwent Claisen rearrangement to furnish **62**. A series of transformations then led to **66**. The latter underwent stereoselective (7:1) epoxidation of the vinyl ether, enabling the creation of the correct configuration of α-benzoyloxy ketone **67**. The subsequent hydration of the ethynyl segment was complicated by loss of the TBS group and consequent formation of hemiketal **68**. Fortunately, **68** smoothly progressed to **69** (Scheme 12) upon enol silylation of the methyl ketone (TBSOTf/*i*Pr_2_NEt) and protection of the primary OH group (TBSCl/imidazole). The crucial aldol-dehydration step was performed in two stages. Reaction of **69** with TBAF induced selective release of the enol silyl ether and consequent aldol addition of the transient enolate to the highly electrophilic α,α′-dioxygenated ketone. Dehydration of the resultant aldol with Cl_3_CCOCl in pyridine delivered **70**. The installation of the nitrogen functionality followed a somewhat circuitous pathway. This was dictated by the instability of the bis-allylic alcohol produced upon reduction of **70** and its protected variants. After much experimenation, it transpired that BOM-protected compound **71** is amenable to conversion into **73**, which, in principle, could undergo an Overman imidate rearrangement as a means to introduce the requisite nitrogen substituent. Unfortunately, all attempts in that sense met with failure. Consequently, **73** was advanced to carbamate **75**, which smoothly cyclized under basic conditions to furnish **76**. The subsequent conversion of **76** into **79** proceeded uneventfully.

**Scheme 13:**
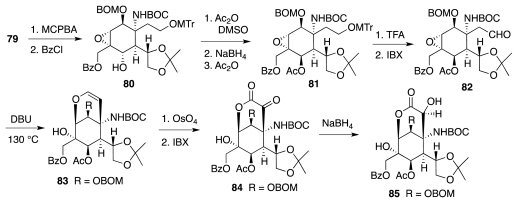


Scheme 13 details the sequence that served to transform **79** into lactone **85**. Points of interest here include the formation of bicyclic vinyl ether **83** through nucleophilic opening of an epoxide by the *O*-terminus of the enolate of an aldehyde (cf. **82**), and the stereoselective osmylation of **83** from the α-face of the vinyl ether. The latter step led to the incorrect configuration of the newly installed non-acetalic OH group, requiring an inversion of configuration by a redox technique (cf. **83**→**85**).

**Scheme 14:**
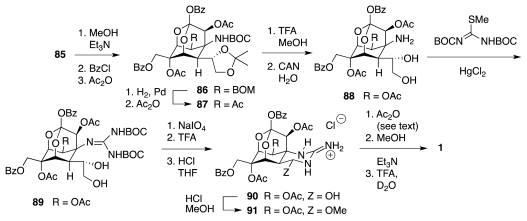


The endgame of the synthesis (Scheme 14) involved a series of carefully orchestrated protection/deprotection steps that prepared the molecule for installation of the guanidine fragment and formation of the complete TTX framework. This led to extremely polar compound **91**, which could be purified only after peracetylation. Final release of all blocking groups completed the first enantioselective synthesis of tetrodotoxin. This monumental tour-de-force reached **1** from **44** in a total of 68 steps, nearly half of which were protection/deprotection maneuvers. Still, the overall yield of TTX was quite respectable (more than 95% yield per step on average).

**Scheme 15:**
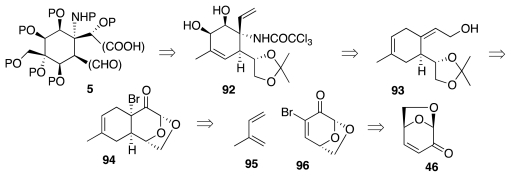


**Scheme 16:**
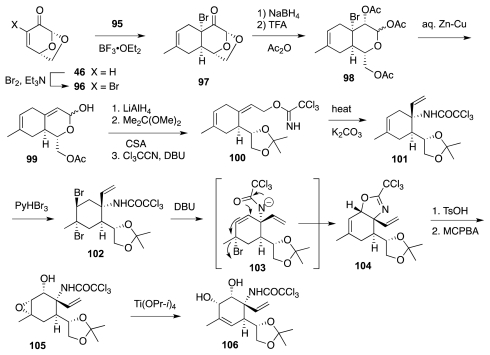


**Scheme 17:**
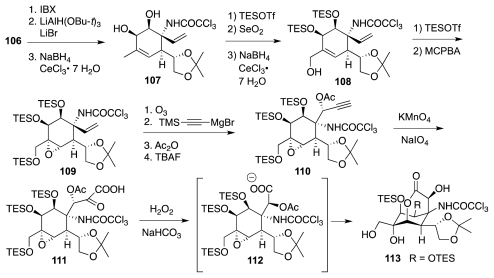


It was alluded earlier to the fact that **73** failed to undergo Overman rearrangement. The 2004 Isobe synthesis (retrosynthetic diagram of Scheme 15) circumvented such a difficulty by carrying out the reaction on a less highly oxygenated substrate, and it introduced an additional refinement in the form of a more convergent assembly of the six-membered ring of the ultimate **5** by a Diels-Alder reaction of isoprene, **95**, with 2-bromoglucosenone, **96**. The major product thus obtained was adduct **97** (Scheme 16). This material was elaborated to **100**, Overman transposition of which afforded **101**. The system was now ready for the installation of the requisite oxygen functionalities. Two interesting transformations were employed in that connection. First, DBU treatment of dibromide **102** (the product of *trans*-diaxial addition of Br_2_ to **101**) induced a tandem E2/S_N_2′ reaction that led to oxazoline **104**. Second, epoxide **105**, prepared by stereoselective *syn*-epoxidation of the allylic alcohol emerging from the hydrolysis of **104**, was rearranged to **106** using Ti(OPr-*i*)_4_. However, the syn-diol system in **106** has the incorrect configuration of relative to **1**. This problem was resolved in the next stage of the synthesis (Scheme 17) by oxidation to a vicinal dicarbonyl and diastereoselective reduction to the correct diol, **107**, which was then transformed into epoxide **109**. Theoretically, the vinyl group in **109** could be oxidatively converted into the requisite α-hydroxyacid branch of **5**. Instead, the authors opted for ozonolysis of **109** and stereoselective (4:1) addition of an acetylide ion to the transient aldehyde; a process that ultimately surrendered **110**. Further oxidative degradation of the acetylene afforded an epoxyacid (cf. **111**), which cyclized spontaneously to yield **113**. Both acetyl and primary TES blocking groups were lost in the course of the latter step.

**Scheme 18:**
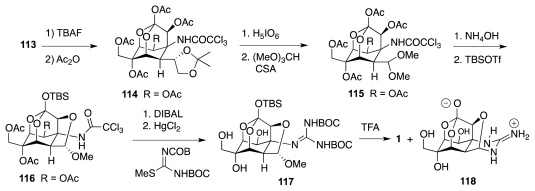


The final attack on **1** ran into some annoying protecting group complications. First, the TES units in **103** provided an inadequate level of OH protection during subsequent manipulations, necessitating a protecting group exchange (cf. **113**→**114**, Scheme 18) prior to oxidative cleavage of the 1,3-dioxolane moiety and protection of the resultant aldehyde as dimethyl acetal **115**. Second, it transpired that the release of the trichloroacetyl segment required TBS protection of the apical OH group. Accordingly, the “upper” acetyl groups were selectively released by exposure of **115** to NH_4_OH. However, silylation of the resultant diol (reaction with TBSOTf) afforded cyclic acetal **116** instead of the desired bis-TBS ether derivative. The synthesis was completed from **116**, which is a structural congener of anhydrotetrodotoxin, **118**, and that indeed produced a mixture of **1** and **118** upon acid treatment.

**Scheme 19:**
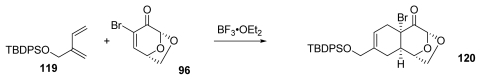


Overall, 37 steps were needed to reach **1** from **46**, only 8 of which entailed protecting group manipulations. Isobe’s second synthesis constitutes a major improvement over the first one and indeed, it lends itself to further amelioration. To wit, a recent paper from the same group describes the Diels-Alder reaction of **96** with oxygenated isoprene **119**, leading to **120** (Scheme 19) [46]. This refinement alone removes 3 steps from the earlier route.

**Scheme 20:**
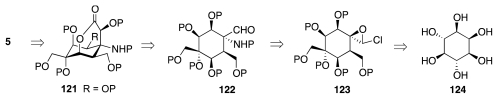


**Scheme 21:**
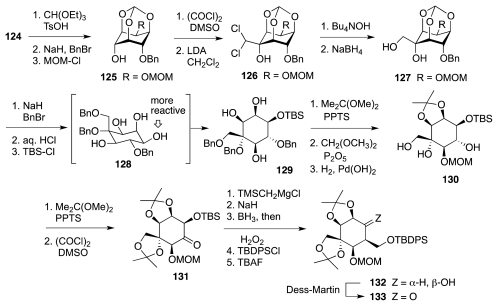


**Scheme 22:**
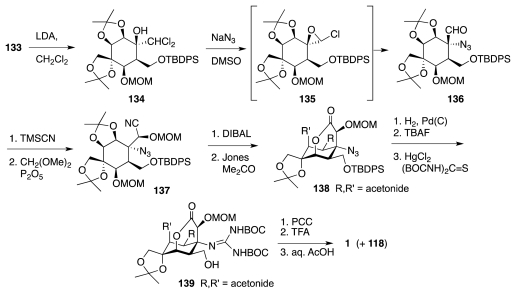


Sato and collaborators have also actively researched avenues to **1** from carbohydrates and related substances. Their first major success was the synthesis of racemic TTX from (achiral) *myo*-inositol [47]. Drawing upon their own work in the carbohydrate field, they considered that a key step toward lactone **121**, an analog of the Isobe intermediate **113**, could be the nucleophilic opening of chloroepoxide **123** with azide ion (Scheme 20). This reaction takes place preferentially at the non-halogenated carbon. The resultant aldehyde **122** would be advanced to **121** via a cyanohydrin, while **123** itself could be manufactured from *myo*-inositol, **124**. The first objective to that end was to outfit the cyclitol for the introduction of the missing carbon branches, two of which were installed through the nucleophilic addition of Li–CHCl_2_ to appropriate carbonyl groups. This required various protection-deprotection steps (Scheme 21), worthy of note among which is the selective (3:1) silylation of the least hindered equatorial alcohol in **128** over the neighboring axial OH. The resultant **129** was then advanced to ketone **133** as a prelude to a second round of Li–CHCl_2_ addition as shown in Scheme 22. Product **134** of this step reacted with NaN_3_ in DMSO containing 15-crown-5 to form a transient epoxide **135**, which in accord with previous studies by the same group suffered *in situ* nucleophilic opening to afford aldehyde **136**. In a fashion somewhat reminiscent of the earlier Isobe synthesis, the latter was homologated to cyanohydrin **137**, which was obtained in a moderately stereoelective manner (1.5:1). A singular mode of lactone formation involved reduction of the nitrile to an aldehyde and treatment of the resultant with Jones reagent in acetone. Evidently, the acidic nature of the oxidant caused release of the MOM group, formation of a hemiacetal, and oxidation to the ultimate **138**. This substance was readily elaborated to (±)-**1** accompanied by some **118**. Sato thus employed 33 steps, 15 of which were protection/deprotection maneuvers, to prepare **1** from **125**.

**Scheme 23:**
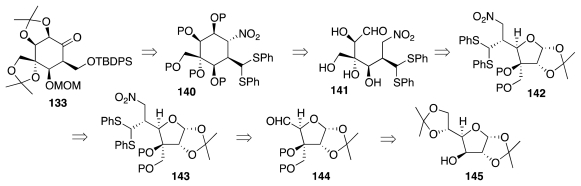


**Scheme 24:**
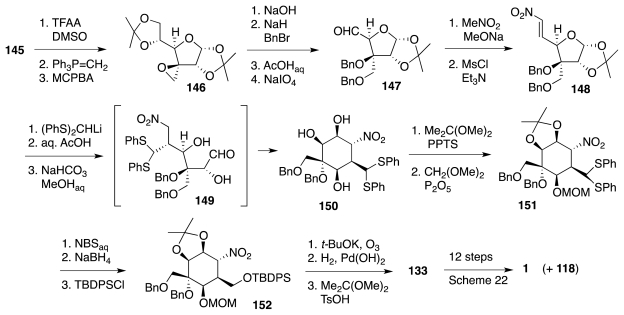


As an alternative to the chemistry of Scheme 21, the same researchers investigated a route to ketone **133** that proceeds through a nitro-aldol (Henry) cyclization of enantiopure compound **141** and Nef reaction of the ensuing **140** (Scheme 23). Compound **141** may be further correlated with glucose diacetonide, **145**, on the basis of an earlier study by Funabashi and collaborators [48]. The implementation of this new approach involved the processing of **145** to **148**, which then underwent diasteroselective (10:1) conjugate addition of lithiated bis(phenylthio)-methane (Scheme 24) [49]. Release of the anomeric acetonide unveiled aldehyde **149**, gentle base treatment of which triggered a highly stereoselective cyclization to **150**. This material was further elaborated to **133**, which as seen earlier in Scheme 22 may be converted into (−)-**1** in 12 steps. In terms of efficiency, the new sequence is comparable to the previous one (33 steps to **1** from glucose, 10 protection/deprotection events).

**Scheme 25:**
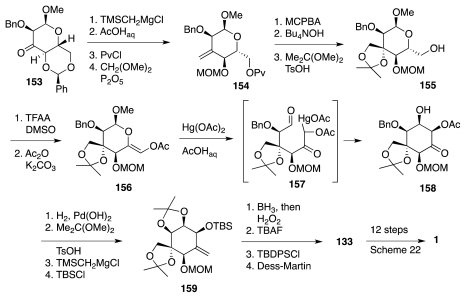


Additional work [50] defined yet another route to **133** from glucopyranose derivative **153** (Scheme 25), available in 4 steps from the parent hexose. Thus, compound **154**, was processed to enol acetate **156**, setting the stage for a crucial Ferrier aldol reaction that afforded the desired **158** as the major component of a 6:2:1 mixture of 3 diastereomers. This material was then elaborated to **133** in a straightforward fashion. In terms of number of steps, this third route (34 from glucose, 12 protecting group manipulations) is also comparable to the previous one.

**Scheme 26:**
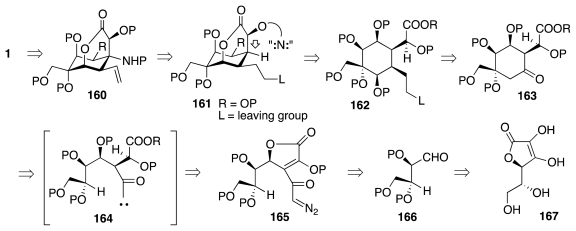


The syntheses just reviewed rely largely on well-established chemical transformations. This begs the question of whether greater conciseness might be achievable though the use of more advanced reactions. A relevant example is apparent in a synthesis of **1** reported by DuBois and collaborators in 2003 [51]. These researchers have been interested in the formation of C–N bonds via nitrenoid insertion into C–H linkages: a process that parallels the analogous reaction of carbenes, but that has not been explored as extensively. Recalling that such insertions occur with rigorous retention of configuration, a retrosynthetic plan toward **1** emerged as sketched in Scheme 26. Lactone **160**, a relative of **113** (Isobe) and **121** (Sato), could ensue through the intramolecular insertion of a nitrene into the appropriate bridgehead C–H bond of **161**. Furthermore, lactone **161** could derive from cyclohexane **162**, a congener of **5** that should be available from ketone **163**. The latter could be manufactured by cyclization of carbene **164** through C–H insertion. The precursor of reactive intermediate **164** would be diazoketone **165**, which appears to be the resultant of the reaction of protected threose **166** with an appropriate nucleophile. A convenient form of the requisite tetrose may be accessed from isoascorbic acid, **166**, which thus becomes the starting point of the synthesis.

**Scheme 27:**
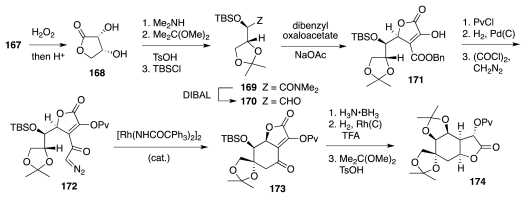


The known oxidative cleavage of **167** to **168** (Scheme 27) served as a prelude to the manufacture of aldehyde **170**. The condensation of the latter with dibenzyl oxaloacetate occurred with significant Felkin-Anh selectivity (>10:1) to furnish lactone **171**, which was then processed to **172**. The carbenoid produced upon reaction of **172** with a Rh(II) catalyst underwent smooth C–H insertion to provide **173**. In accord with solid precedent, the subsequent reduction of the ketone and hydrogenation of the double bond occurred both selectively from the α-face of the molecule. Interestingly, by carrying out the hydrogenation step under acidic conditions, the saturated form of **173** underwent *in situ* release of the TBS group and lactone isomerization, predisposing the molecule for reprotection of the liberated diol as an acetonide. This delivered advanced intermediate **174**.

**Scheme 28:**
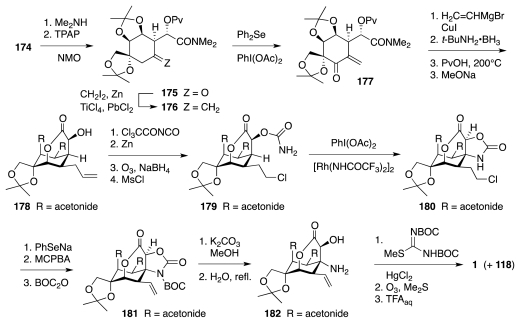


Carbamate **179** (Scheme 28) was subsequently prepared from **174** via a straightforward sequence of transformations. This late intermediate underwent a remarkable oxidative cyclization to **180** upon treatment with iodobenzene diacetate and Rh(II) trifluoroacetamidate. With **180** in hand, the synthesis of **1** was completed quickly. Overall, 34 steps were necessary to prepare **1** from **167** (28 from **170**), but only six of these were true protecting group manipulations.

### Synthetic Studies toward TTX Based on Carbohydrate Building Blocks and Congeners

**Scheme 29:**
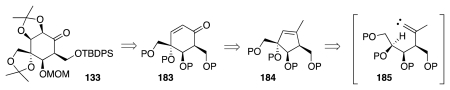


In addition to the above syntheses, the literature records a significant volume of research focusing on the construction of tetrodotoxin precursors from naturally occurring, polyhydroxylated substances. Some of these studies have explored strategically interesting principles. For instance, an approach disclosed by Taber, which appeared in print just days after the publication of DuBois’ work, addressed the preparation of enone **183** through a C–H insertion reaction of an alkylidene carbene, according to the format of Scheme 29 [52]. Compound **183** is a plausible forerunner of the Sato intermediate, **133**.

**Scheme 30:**
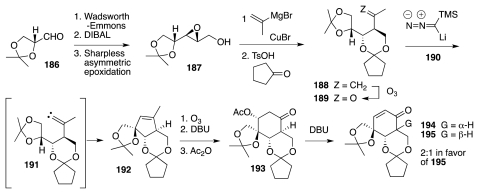


Convenience advocated investigating the feasibility of this approach using compounds possessing the antipodal configuration. In fact, *ent*-**183**, rendered in Scheme 30 as compound **195**, appeared to be readily accessible from (d)-glyceraldehyde acetonide, **186**. The latter thus was transformed into ketone **189**, which upon reaction with lithiated TMS-diazomethane, **190**, underwent Peterson-type olefination to a highly unstable diazo compound. The latter suffered spontaneous deazoniation to vinylidene **191**. This reactive intermediate efficiently inserted into the proximal C–H bond of the dioxolane moiety to form **192**. A subsequent ozonolysis-aldol-acetylation sequence gave **193**, which relative to *ent*-**183** possesses the incorrect configuration of the stereogenic center adjacent to the carbonyl group. Prolonged treatment with DBU induced both elimination of the acetate and equilibration to a 2:1 mixture of **195** (desired cis-diastereomer, major) and **194**.

**Scheme 31:**
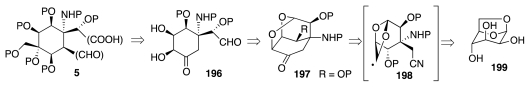


Also worthy of note is an approach to **1**, described by Fraser-Reid [53–55], which rests upon the surmise adumbrated in Scheme 31. Retrosynthetic simplification of **5** leads to aldehyde **196**, and thence to tricyclic acetal **197**. An interesting construction of the latter envisions the formation of the cyclohexanone segment through the addition of a carbon radical to a nitrile (cf. **203**). Structure **198** may be correlated with an anhydrohexose, e.g., (d)-mannosan, **199**.

**Scheme 32:**
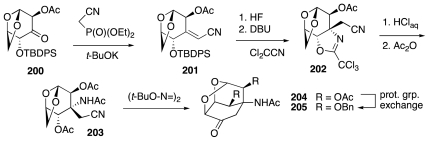


These ideas were translated into practice starting with the known mannosan-derived ketone **200** (Scheme 32). The corresponding product **201** of Wadsworth-Emmons olefination was desilylated and converted into a trichloroacetimidate, which then underwent Michael cyclization to afford **202** in a fashion that presaged a similar transformation later employed by Isobe in his own synthesis of **1**. The substrate for the key radical cyclization was **203**, which upon exposure to bis-*tert*-butyl hyponitrite in refluxing *tert*-butanol surrendered tricyclic ketone **204**. Evidently, a *tert*-butoxy radical ensuing upon thermolysis of the hyponitrite preferentially abstracted a hydrogen atom from the oxymethylene bridge of **203**, forming a radical such as **198**, which then added to the nitrile. A blocking group exchange then yielded benzyl-protected derivative **205**.

**Scheme 33:**
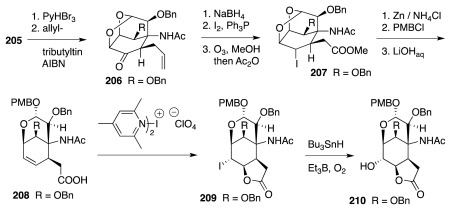


Relative to **196**, compound **205** lacks the future aldehyde functionality. The next phase of the work (Scheme 33) thus targeted Kishi-type intermediate **210**: a congener of substance **37** in Scheme 6. A key step in the preparation of **210** was a radical allylation of the bromo derivative of **205**. The construction of **210** was completed by reductive cleavage of bicyclic acetal **207**, iodolactonization of acid **208** by the use of bis(collidine)iodonium perchlorate, and a final radical oxygenation. Compound **210** represents the most advanced construct yet disclosed in this series.

**Scheme 34:**
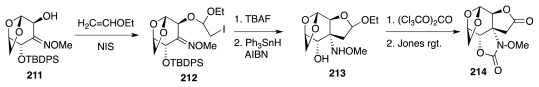


Under the rubric of radical reactions, Alonso described a route to lactone **214** (Scheme 34), which is an analog of nitrile **202**. Access to **214** was secured from **211**, recognized as a deacetylated *O*-methyl oxime derivative of **200**, by formation of a 2-iodoacetal from the free alcohol, cyclization with Bu_3_SnH/AIBN, protection, and Jones oxidation [56,57].

**Scheme 35:**
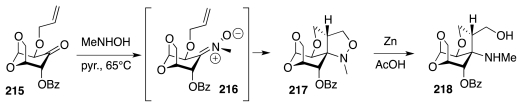


The same group also explored the assembly of compounds structurally related to **214** via an intra-molecular cycloaddition of carbohydrate-based nitrones. Thus, reaction of mannosan derivative **215** with *N*-methylhydroxylamine hydrochloride occasioned the formation of a nitrone (cf. **216** or its geometric isomer, Scheme 35), which cyclized directly to isooxazolidine **217** [58,59]. Reduction of the N–O bond produced **218**, thereby demonstrating an interesting method for the creation of nitrogen-bearing tetrasubstituted carbon centers.

**Scheme 36:**
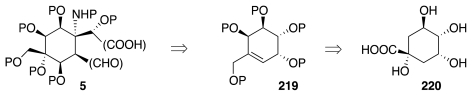


Finally, Ohfune researched an avenue to compound **5** not from a carbohydrate, but from quinic acid, **220**, by way of cyclohexene **219**, according to the format of Scheme 36.

**Scheme 37:**
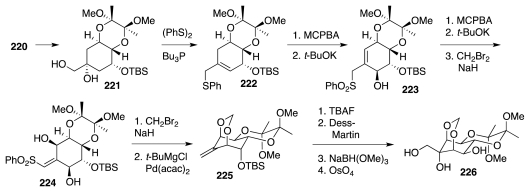


Thus, **220** was elaborated to **221**, which underwent simultaneous substitution of the primary OH group and regioselective dehydration of tertiary alcohol upon reaction with (PhS)_2_ and Bu_3_P (Scheme 37). The action of MCPBA on the emerging **222** resulted in oxidation of the sulfide to the sulfone and selective epoxidation of the olefin from the β-face. Subsequent base treatment caused eliminative fragmentation of the epoxide orchestrated by the sulfonyl group along with deconjugation of the exomethylene sulfone, thus formed to the more favorable isomer **223**. A second round of epoxidation/elimination produced **224**, which was protected and reductively desulfonylated to afford **225**. The latter was then advanced to **226**, which represents the terminus of this synthetic study [60].

## 5. Other Approaches to TTX through Diels-Alder Reactions or Annulation Processes

**Scheme 38:**
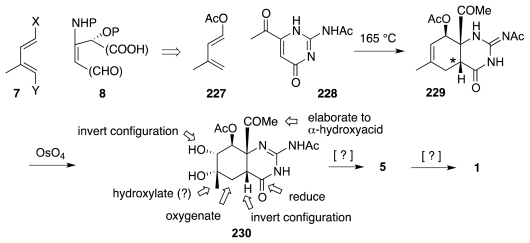


In the 1970s and early 1980s, Keana investigated a unique approach that targeted an early intermediate already incorporating the guanidine unit [61–63]. This strategy rested on the use of pyrimidinone **228** as an unusual dienophile *vis-a-vis* diene **227** (Scheme 38). The more electrophilic COMe group controlled the relative orientation of the reactants, which combined to afford adduct **229** as the major product. Relative to **5**, this material possesses the incorrect configuration at the starred center, but the adjacent carbonyl group might permit epimerization to the correct, and presumably thermodynamically favored, trans-fused diastereomer. This isomerization, however, remains to be demonstrated (or disclosed).

An important step that was indeed described is the osmylation of **229**, a reaction that occurred with essentially no facial selectivity. The α-diastereomer of the *syn*-diol (**230**, desired) and its β-isomer were separated by column chromatography. Diol **230** constitutes Keana’s most advanced TTX intermediate yet reported. Its elaboration to **1** could involve the operations listed in Scheme 38; but these conjectures remain to be reduced to practice.

**Scheme 39:**
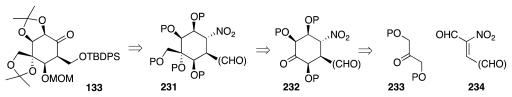


In 2010 Alonso described a construction of nitroketone **232** (Scheme 39) through annulation chemistry [64]. Compound **232** is recognized as a precursor of the Sato TTX intermediate, **133** (cf. Scheme 21), and it was regarded as resulting via the [3 + 3] union of protected dihydroxyacetone, **233**, with a nitroolefin such as **234**.

**Scheme 40:**
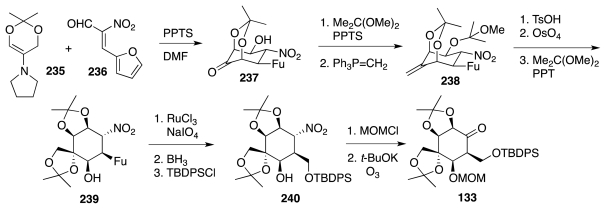


Suitable forms of components **233** and **234** proved to be, respectively, the pyrrolidine enamine of the isopropylidene derivative of dihydroxyacetone, **235**, and furyl nitroalkene **236** (Scheme 40). When admixed in DMF in the presence of PPTS, these combined to afford (racemic) **237** as the major product in a highly diastereoselective manner. This material was advanced to nitrocyclitol **239**, which required no protection of the free OH group during oxidative degradation (catalytic RuO_4_) of the furyl group to a carboxylic acid. Reduction of the latter, differential OH protection and Nef reaction as detailed earlier by Sato produced (±)-**133**. Given that Sato had also described the conversion of enantiopure **133** into (−)-TTX (12 steps, *vide supra*), the preparation of (±)-**133** amounts to a formal synthesis of (±)-**1**.

## 6. Approaches to TTX that Rely on Intramolecular Nitrile Oxide Cycloaddition Reactions

**Scheme 41:**
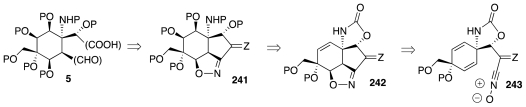


The CHO and the adjacent OP group in compound **5** constitute an aldol motif. Among the ways in which such a feature could be created, noteworthy is the [3 + 2] cycloaddition of a nitrile oxide to an olefin, followed by cleavage of the resulting oxazoline [65,66]. In the specific case of **5** (Scheme 41), the *syn* relationship of the “southeastern” OP, CHO and COOH functional triad evokes a possible intramolecular nitrile oxide cycloaddition (INOC), wherein the reactive dipole is appended to that branch of the substrate that is destined to become the hydroxyacid segment of **5**. Group Z in retrosynthetic intermediate **241** represents a functional ensemble that would ultimately enable the fragmentation of the oxazoline unit. Further analysis reveals that an appropriate conformational constraint might even permit the conduct of the INOC step on a “locally symmetrical” cyclohexadiene such as **243**. Such a strategy may translate into an especially concise synthesis.

**Scheme 42:**
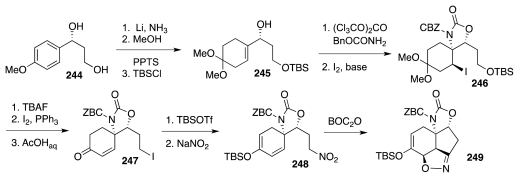


In 2002, Fukuyama and coworkers disclosed the first study ever to address the foregoing surmise [67]. The intial subgoal of their effort was nitro compound **248** (Scheme 42), which was prepared starting with a Birch reduction of **244**, readily available in turn from *p*-anisaldehyde. A straightforward sequence advanced the Birch product to an *N-*CBZ carbamate, in preparation for iodocyclization to **246**. Elaboration of this substance to a diiodo derivative, followed by release of the ketal, triggered elimination of HI from the nascent β-iodoketone and afforded enone **247**, which then progressed to **248**. Treatment of the latter with BOC_2_O served to induce dehydration of nitro group to a nitrile oxide and consequent formation of **249**.

**Scheme 43:**
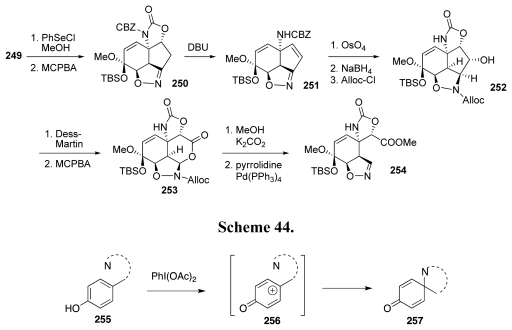


The next stage of their endeavor centered on the production of isoxazoline **254** (Scheme 43). To that end, **249** was first transformed into mixed ketal **250**. The oximino unit in **250** activates the neighboring methylene toward deprotonation. Indeed, exposure of **250** to DBU promoted decarboxylative β-elimination of the oxazolidinone to give **251**. A subsequent reaction with OsO_4_ took place selectively at the more exposed and more strained cyclopentene double bond and the nascent diol cyclized to a new oxazolidinone, **252**. Some redox adjustments prepared the molecule for a Bayer-Villiger oxidation reminiscent of the Kishi synthesis (cf. **35**, Scheme 6) and leading to lactone **253**. Two more steps afforded the target **254**, in which the isooxazoline constitutes a protected form of the β-hydroxyaldehyde segment of **5**.

**Scheme 44:**
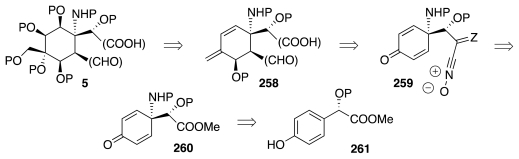


The foregoing outline of the various approaches to TTX provides the background for a summary of our own research in this domain. Our group has developed a suite of chemical transformations that may be collectively described as the “oxidative amidation” of phenols, and that may be represented with the general diagram of Scheme 44 [68–70]. Thus, oxidative activation of a phenol such as **252**, typically with a hypervalent iodine reagent like PhI(OAc)_2_, produces a reactive intermediate, which we ideate as cation **256**. Substituent N represents an appropriate nitrogen nucleophile, while the dashed semicircle signifies that the “N” may be tethered to the phenolic nucleus, or it may be independent. In the first case, the reaction occurs in an intramolecular fashion; in the second, in a bimolecular regime. In all cases, “N” emerges from the reaction as part of an amide functionality; hence the terminology “oxidative amidation of phenols”. Three modes of oxidative amidation are currently known, depending on whether N is part of an oxazoline (intramolecular) [71–73], a sulfonamide or a phosphoramide (intramolecular) [74–76], or a nitrile (bimolecular) [77,78]. The reaction is most often carried out with *para*-substituted phenols, as dictated by the structure of various synthetic targets [79–81], but *ortho*-oxidative amidation is perfectly possible [70,75,82].

**Scheme 45:**
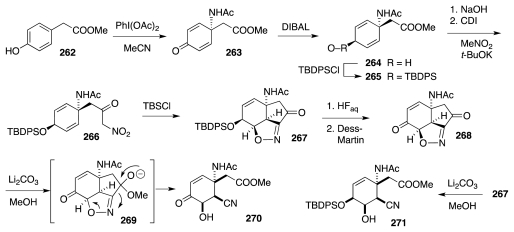


The nexus between oxidative amidation technology and TTX emerges from the formulation of Scheme 45. In principle, **5** could be made by double osmylation of **258**. If one envisions a Wittig reaction as a means to create the exomethylene system and an INOC step to introduce OP and CHO groups, then **258** simplifies to **260**, which is the product of oxidative amidation of **261**. The hypothesis of Scheme 45 is one of the reasons why the senior author of this review launched a program aiming to establish a bimolecular phenolic amidation reaction beginning in the late 1990s. The advent of suitable methodology [77,78] served to verify that a substrate of the type **261**, P = Me, indeed undergoes oxidative amidation in good yield [82].

**Scheme 46:**
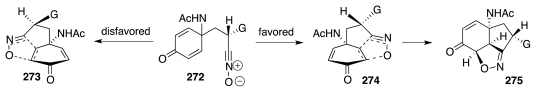


It seems superfluous to state that the full implementation of the above logic requires considerable amounts of exploratory work. Initial feasibility studies centered on the crucial INOC step in a desoxy series emanating from ester **262** (Scheme 46). Oxidative amidation to **263**, chemo- and diastereoselective reduction of the ketone, and protection of the OH group set the stage for saponification of the ester and creation of nitroketone **266**. Treatment of the latter with TBSCl and imidazole induced a Torssell-like cyclization to (±)-**267**, which was then elaborated to (±)-**268**. The action of methanolic Li_2_CO_3_ on (±)-**268**, induced formation of (±)-**270**, arguably through fragmentation of hemiketal anion (±)-**269**. In a like fashion, compound (±)-**267** itself was advanced to (±)-**271** [83].

**Scheme 47:**
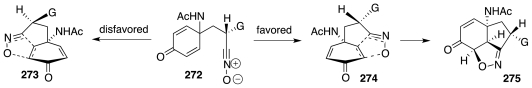


This chemistry provided proof for the principle adumbrated in Scheme 45; however, in the form presented above it only provides racemic materials. An enantiocontrolled variant may be possible if the nitrile oxide arising through dehydration of **266** could be directed to one of the two diastereotopic double bonds of the dienic segment. To that end, Fukuyama had opted to tether nitrogen and oxygen functionalities as evident from intermediate **248** of Scheme 42. For reasons that will be become apparent shortly, we favored the alternative outlined in Scheme 47. Nitrile oxide **272** is a congener of **259** that carries a sterically demanding group G of the indicated configuration. This substituent would also have to function as the forerunner of a carbonyl group, enabling the eventual fragmentation of the isoxazoline as seen earlier in Scheme 46. It seemed likely that the steric bulk of G would cause **272** to undergo INOC cyclization preferentially through transition state **274**, wherein G resides outside the developing bowl-shaped tricyclic product throughout the reaction. By contrast, transition state **273** would force G within the developing bowl, creating severe steric congestion. If so, the INOC step should involve preferentially the *pro*-(*S*) double bond of **272**, resulting in selective formation of **275**. The configuration of G would thus determine that of three other stereocenters, including that of the nettlesome tetrasubstituted carbon bearing the NHAc group.

**Scheme 48:**
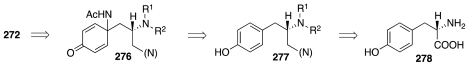


If G were a bulky nitrogen functionality, then a suitable precursor of **272** could be manufactured from (l)-tyrosine, according to Scheme 48 (cf. NR^1^R^2^ in **276**). In this formulation, (N) represents a second nitrogenous functionality amenable to conversion into a nitrile oxide at an appropriate juncture. An interesting opportunity materializes when one considers possible (N) groups that could advance to nitrile oxides under the same conditions employed for the oxidative amidation step; *i.e.*, upon treatment with hypervalent iodine reagents: the two steps could carried out in tandem, provided that the rate of oxidation of (N) to a nitrile oxide were slower than that of the oxidative amidation step. This condition finds justification in the fact that the dienone system must be already in place by the time that the nitrile oxide begins to form; otherwise, the INOC step could not occur. In the event, it transpired that group (N) had to be an aldoxime [84–86].

The vision of Scheme 48, was translated into reality starting with the derivatization of (l)-tyrosine to **279**, which upon reaction with PhI(OCOCF_3_)_2_ in acetonitrile afforded tricyclic product **280** as the sole detectable diastereomer (Scheme 49). A compound of such a complexity thus emerged in a mere 5 steps from a common aminoacid. Efforts are currently underway to complete a total synthesis of TTX from one of these advanced intermediates.

## 7. Conclusion

The discussion just completed conveys a sense of why synthetic research on tetrodotoxin remains as active as it is. The development of TTX-like drug requires a great deal of medicinal chemistry work, which in turn becomes realistically possible only upon the advent of suitable synthetic technology. Progressive refinements in the latter domain will hopefully enable a full-out effort in the former before too long. It is this synergy between medicine and chemistry that has traditionally driven advances in the science of synthesis, which remains as central as ever as a source of new leads for the creation of better therapeutic agents.

## Figures and Tables

**Scheme 49:**